# Electric toothbrush application is a reliable and valid test for differentiating temporomandibular disorders pain patients from controls

**DOI:** 10.1186/1471-2474-10-94

**Published:** 2009-07-30

**Authors:** Donald R Nixdorf, Azar Hemmaty, John O Look, Eric L Schiffman, Mike T John

**Affiliations:** 1 Department of Diagnostic & Biological Sciences, School of Dentistry, Minneapolis, MN, 55455, USA; 2 Department of Neurology, Medical School, University of Minnesota, Minneapolis, MN, 55455, USA; 3 Department of Epidemiology, School of Public Health, University of Minnesota, Minneapolis, MN, 55455, USA

## Abstract

**Background:**

Current methods for identifying patients with pain hypersensitivity are sufficiently complex to limit their widespread application in clinical settings. We assessed the reliability and validity of a simple multi-modal vibrotactile stimulus, applied using an electric toothbrush, to evaluate its potential as a screening tool for central sensitization.

**Methods:**

Fourteen female temporomandibular disorders (TMD) subjects with myofascial pain (RDC/TMD Ia or Ib) and arthralgia (RDC/TMD IIIa) were compared to 13 pain-free controls of matched age and gender. Vibrotactile stimulus was performed with an electric toothbrush, applied with 1 pound pressure for 30 seconds in four locations: over the lateral pole of the temporomandibular joint, masseter, temporalis, and mid-ventral surface of forearm. Pain intensity (0–10) was recorded following the stimulus at 0, 15, 30, and 60 seconds. Test-retest reliability was assessed with measurements from 8 participants, taken 2–12 hours apart. Case versus control differentiation involved comparison of area under the curve (AUC). A receiver operating characteristic (ROC) curve was used to determine cutoff AUC scores for maximum sensitivity and specificity for this multi-modal vibrotactile stimulus.

**Results:**

Test-retest reliability resulted in an ICC of 0.87 for all 4 pooled sites. ROC-determined AUC cutoff scores resulted in a sensitivity of 57% and specificity of 92% for all 4 pooled sites.

**Conclusion:**

The electric toothbrush stimulus had excellent test-retest reliability. Validity of the scores was demonstrated with modest sensitivity and good specificity for differentiating TMD pain patients from controls, which are acceptable properties for a screening test.

## Background

Central sensitization, also known as hypersensitivity, seems to be a common feature of chronic pain conditions [1]. In patients with painful temporomandibular disorders (TMD), pain is associated with centrally-mediated sensitization, as measured by several quantitative sensory testing (QST) modalities [2-5]. In general, clinicians and researchers active in this area agree that compared to pain-free patients, people with chronic TMD pain are more likely to have alterations in their central processing of external stimuli within the structures innervated by the trigeminal nerve, resulting in lower sensory thresholds [6,7]. This is evidenced by alterations in measures of TMD pain patients' static pressure-pain threshold [5,8], vibrotactile stimulation perception [4], and noxious heat threshold [9,10], and by the results of dynamic suprathreshold tests involving temporal summation, such as the sub-maximal ischemic tourniquet test [2,3] and the cold-pressor test [11]. Similar findings have been reported for other chronic orofacial pain conditions, including chronic tension-type headaches [12] and neuropathic trigeminal pain [13-15].

At present there is no standard clinical measure for central sensitization. Several methods of applying QST for a variety of stimuli have been reported [16,17], but their application in clinical practice has largely been restricted to the diagnoses of neuropathic pain [18-20]. Development of a simple, robust test to identify people with hypersensitivity may be advantageous to healthcare providers. The ability to readily differentiate people with increased sensitization could have implications for the diagnostic testing and classification of pain disorders, and for the prognostication of chronic pain treatments and patients' post-operative pain experience [1].

Use of an electric toothbrush to apply a vibrotactile stimulus may produce the desired attributes of not only dynamic mechanical stimulation, but also thermal and punctuate mechanical stimulation, with temporal summation. Such a multi-modal stimulus device has many practical advantages that increase its likelihood of being incorporated into clinical practice: The device is low cost, small and easy to store, simple to operate, and readily available for purchase. In addition, the stimulation technique is not time consuming (requires less than two minutes per stimulus site), is known to dentists, and is well tolerated by subjects. For these reasons, an electric toothbrush it is a promising instrument for use in a potential screening test for central sensitization.

One of the first steps in developing a screening tool of this type is to assess the tool's reliability and groups validity, i.e., its ability to distinguish a population with known susceptibility to central sensitization (such as chronic TMD pain patients) from pain-free controls. Groups validity testing is an essential part of construct validity assessment [21-23]. Therefore, the aims of this study were to determine the test-retest reliability of a vibrotactile stimulus delivered with an electric toothbrush and to evaluate its validity in differentiating people with TMD pain from pain-free controls. This work is an important initial step towards the development of a screening tool for routine clinical use.

## Methods

The research protocol was reviewed and approved by the University of Minnesota's Institutional Review Board. Before providing written consent and HIPAA authorization, potential participants were given an explanation of the study and the opportunity to ask questions about the protocol.

### Subjects

Fliers and newspaper advertisements were placed around the University of Minnesota campus and in University clinics to recruit TMD pain cases and pain-free control subjects, matched on age and gender. Women between 18 and 60 years old and who were not pregnant or lactating were invited to be screened for study eligibility.

To be included as cases, women had to have self-reported pain in both the masseter muscle and temporomandibular joint (TMJ) over the last month, with a pain intensity in the range of 3 to 8 on a 10-point scale (with 0 representing "no pain" and 10 representing the "worst pain imaginable"). In addition, they had to meet the diagnostic criteria for myofascial pain and arthralgia (RDC/TMD Ia or Ib and IIIa) [24], as rendered by a calibrated clinical examiner. Pain had to be present at the time of testing and at least 50% of the time, which is consistent with the International Classification of Headache Disorders [25]. Pain also had to be present for at least 6 months prior to the sensory testing, but no longer than 10 years; this criterion was to ensure that the pain was chronic in nature, while excluding subjects with intractable pain, since they are most likely to have irreversible changes in the central pain pathways. Subjects' pain could be either unilateral or bilateral. If bilateral, vibrotactile stimulation was performed on the most painful side. Signs or symptoms of TMJ disc displacement were allowed, as long as noises that occurred with jaw function did not specifically elicit an increase in pain.

Pain-free controls were defined as women with no history of bodily pain within the past 3 months, no history of jaw pain, and a normal, pain-free mandibular range of motion (*e.g*., 40 mm vertical inter-incisal opening corrected for anterior vertical overlap).

For both controls and cases, we excluded anyone with a major systemic illness related to altered pain sensitivity, or with fibromyalgia or other widespread bodily pains; a history of TMJ surgery or inter-articular steroid injection; a history of traumatic injuries to the orofacial region; currently receiving active TMD treatment; or taking analgesics and other medications that could alter stimulus sensitivity.

### Psychophysical Testing

**Figure 1:**
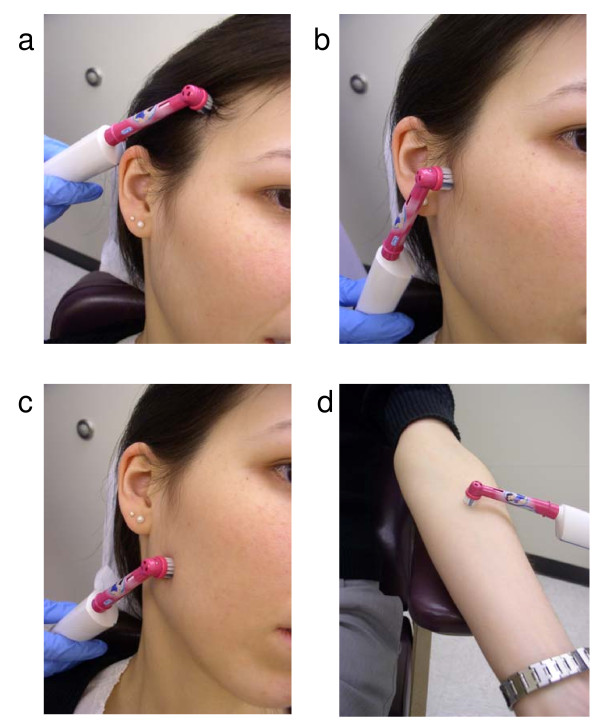
**Example of vibrotactile stimuli being applied at all four sites: a) temporalis muscle, b) lateral pole of temporomandibular joint (TMJ), c) masseter muscle, and d) mid-ventral contralateral forearm**.

Vibrotactile stimulus with an electric toothbrush was performed by one trained person (AH), a dentist who was unaware of subject's pain status. The stimulus was applied during the 5^th ^through 10^th ^days of the subject's menstrual cycle, if a cycle was present, since research suggests that pain sensitivity in women can vary across different stages of the menstrual cycle [11]. The stimulus was delivered by using an electric toothbrush (Braun Oral-B) with the following properties: brush head of 1 cm in diameter, with 22 tufts of bristles and approximately 50 polished bristles per tuft. The head of the toothbrush moved in a rotational manner, 10 degrees in each direction, at a frequency of 5 Hz. The brush head was applied perpendicular to the skin with 1 pound pressure for 30 seconds, or for the maximum time tolerated when less than 30 seconds. Prior to and immediately following application of the stimulus, the examiner calibrated the pressure applied by using a postal scale. The vibrotactile stimulus was applied in four locations (Figure 1). Three were ipsilateral to the self-report of greatest pain: over the lateral pole of the temporomandibular joint, mid-masseter, and anterior temporalis. One site was contralateral to self-report of greatest pain (mid ventral forearm) to serve as a control location (one that is not innervated by the trigeminal nerve), as previously used by others [2]. The contralateral ventral forearm site was included to capture wide-spread hypersensitivity, if present.

**Figure 2:**
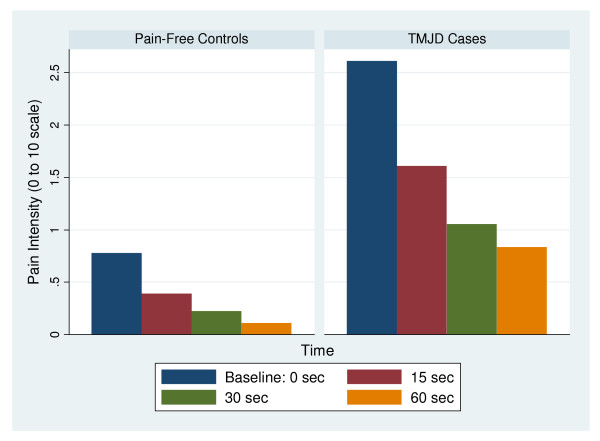
**Mean pain rating for the masseter muscle site at each time point (0, 15, 30, 60 sec) for pain-free controls and TMD cases**.

Resultant pain intensity was measured on a 0–10 numeric rating scale following the vibrotactile stimulus at 0, 15, 30, and 60 seconds. For each individual, the pain intensity figures were plotted against time and connected with a line. The area under the line was calculated and used as the measure of vibrotactile stimulation. Therefore, the area represented the amount of pain experienced by the individual over time, measured in pain intensity-seconds (Figure 2). The maximum area for a single test site was 10, multiplied by 60 seconds, or 600 intensity-seconds. With all four test sites pooled together, the maximum potential area was 2400 intensity-seconds.

This method of psychophysical testing differs from the diagnostic palpation component of the RDC/TMD in four ways. First, the vibrotactile stimulus includes psychophysical modalities other than palpation, such as vibration and brushings of the skin. Second, less pressure is used (1 pound versus the 2 pounds used for muscle palpation. Third, fewer numbers of sites are used (4 as opposed to 20), with one of the sites being outside the innervation of the trigeminal nerve. Fourth, the outcome measure is not a static dichotomous "yes/no" response or a range of "no pain/mild/moderate/severe," but rather a dynamic measure of pain intensity over time, following a standard stimulus duration; this is thought to be the best method to assess wind-up and represents changes in the central modulation of pain [26].

### Reliability Assessment

Test-retest reliability was assessed in a convenience subset of subjects (N = 8) for whom the stimuli were administered twice, over a time interval of 2 to 12 hours. The skin at the site of stimulus application was marked with a felt pen for ease of identification upon reapplication for test-retest reliability. Intraclass correlation coefficients (ICC) and their 95% confidence intervals were calculated based on a one-way repeated measures ANOVA [27]. Limits of agreement were computed around the mean of the differences including a 95% confidence interval for the mean [28]. The goodness of the reliability coefficients was evaluated according to published guidelines [29].

### Validity Assessment

Differentiation of TMD pain subjects from controls is a component of construct validity, known as groups validity [21,22]. Statistical differentiation of TMD pain cases versus pain-free controls involved a receiver operating characteristic (ROC) curve. At each point of the vibrotactile stimulus scale, a two by two table was constructed that tabulated predicted and true TMD case and control status. Sensitivity and 1-specificity were derived from each table. A ROC curve plotted sensitivity versus 1-specificity over the entire range of the vibrotactile stimulus scale. The maximum sum of sensitivity and specificity was determined as the point of the vibrotactile stimulus measure for best differentiation of TMD cases from controls. Confidence intervals for the area under the (ROC) curve (AUC) were determined for each test site and pooled tests to determine the goodness of differentiating cases from controls. An area of 1 represents perfect discrimination, an area of 0.5 represents no discrimination at all, and an area of 0 represents perfect discrimination with an inverse relationship. Goodness of AUC was interpreted according to guidelines .

### Statistical Methods

All analyses were performed using the statistical software package STATA (Stata Statistical Software: Release 9. College Station, TX: StataCorp LP). Differences between cases' and controls' characteristics (age, ethnicity, marital status, level of education, mean household income, presence of any headache in last year, and presence of migraine headaches) were tested using a t-test for continuous data and chi-squared tests for dichotomous variables. Results were considered statistically significant if p < 0.05.

## Results

### Subjects

All TMD pain cases and pain-free control subjects were evaluated over a period of four months at the University of Minnesota Oral Health Clinical Research Center, within the School of Dentistry. Nineteen people with TMD pain were screened to obtain the 14 cases; most were recruited from clinical settings at our institution. Fifteen pain-free controls, matched on age and gender, were screened to obtain the 13 controls. All of those screened who fit the enrollment criteria agreed to participate in the study, and all enrolled subjects completed the research protocol.

Cases and controls were of similar age, marital status, and socioeconomic status (Table 1). A higher proportion of white subjects were observed in the TMD pain group than in the control group. Reports of headache pain in the last year and a diagnosis of migraines were more frequent in TMD pain subjects than in the control group, but these differences did not reach statistical significance, likely due to low power.

**Table 1:** Characteristics of Study Subjects

	TMD pain subjects N = 14	Control subjects N = 13	p-value
	
	*Mean (SE) or N (%)*		
Age, *years*	36 (3.1)	36 (3.3)	0.95

Ethnicity, *white*	13 (93%)	8 (62%)	0.05

Marital status, *married*	5 (36%)	5 (38%)	0.88

Education, *> high school*	13 (93%)	13 (100%)	0.96

Household income, *>$40,000/year*	7 (50%)	8 (62%)	0.55

Headache presence in last year, *yes*	12 (86%)	7 (54%)	0.07

Migraine diagnosis, *yes*	6 (43%)	2 (15%)	0.12

The TMD pain subjects self-reported a mean pain intensity of 4.6 (95% CI 4.0–5.3) over the last month and 3.9 (95% CI 3.0–4.9) on the day of evaluation, prior to vibrotactile stimulation. On average, the time since onset of their TMD pain was 109 months (95% CI 74–143).

All subjects tolerated the vibrotactile stimulus, and no one withdrew from the study. A complete set of data points were obtained (no missing values).

### Assessment of Reliability

Except for one reliability coefficient, all coefficients exceeded the threshold of 0.75 (Table 2), which is considered "excellent" reliability [30]. The most variable stimulus location was the temporalis site, which had an ICC of 0.57. By applying accepted guidelines, this level of reliability can be considered "moderate." The highest reliability coefficient observed was for the masseter site, 0.98, with almost perfect reliability (95% CI 0.96 to 1.00).

**Table 2:** Test-retest Reliability

Site	ICC^1 ^(95% CI*)	Test-retest differences in *intensity seconds*	
		
		Mean (95% CI*)	Limits of agreement
Masseter	0.98 (0.96 – 1.00)	2 (-1 to 5)	-5 to 9
Temporalis	0.57 (0.05 – 1.00)	-19 (-52 to 14)	-91 to 52
TMJ	0.86 (0.67 – 1.00)	-5 (-21 to 12)	-44 to 35
3 TMD sites pooled	0.84 (0.64 – 1.00)	-13 (-55 to 28)	-112 to 86
Forearm	0.79 (0.51 – 1.00)	-5 (-14 to 4)	-26 to 16
All 4 sites pooled	0.87 (0.70 – 1.00)	-24 (-65 to 16)	-122 to 73

^1 ^ICC – intraclass correlation coefficients, * CI – confidence interval

### Assessment of Validity

Three variables (temporalis, three TMD sites combined, and all four sites combined) reached "fair" discrimination of cases from controls. The lower limits of their 95% confidence intervals exceeded the 0.5 value (Table 3), providing evidence for groups validity. The remaining three variables (masseter, TMJ, and forearm) did not reach the threshold of 0.7 for "fair" discrimination. These had AUCs of 0.66 to 0.69, and their lower confidence limits of 0.45 to 0.49 did not reach the 0.5 threshold for statistical significance.

**Table 3:** Differentiating TMD Cases from Controls

Site	AUC* (95% CI)	Best cut-off point (max. no of subjects correctly classified)			
		
		Cut-off point	Correctly classified	Sensitivity	Specificity
			
		*[intensity sec]*	*[%]*		
Masseter	0.69 (0.49 – 0.89)	60	74	57	92
Temporalis	0.77 (0.58 – 0.95)	30	78	71	85
TMJ	0.66 (0.45 – 0.87)	128	67	43	92
3 TMD sites combined	0.77 (0.69 – 0.95)	140	74	57	92
Forearm	0.66 (0.46 – 0.87)	8	70	64	77
All 4 sites combined	0.79 (0.62 – 0.97)	218	74	57	92

* AUC – Area under curve

## Discussion

### Interpretation and Relevance of Findings

Our study suggests that the application of a vibrotactile stimulus using an electric toothbrush is a reliable and valid approach to measuring central pain sensitization in the orofacial region of subjects with TMD pain for screening purposes. This implies that the application of such a stimulus may be able to distinguish people who have increased central sensitization associated with the presentation of trigeminally-mediated TMD pain from those who do not have such pain.

### QST as Assessment for Central Sensitivity

A positive pain response to an individual QST modality is suggestive of sensitization (in the absence of signs or symptoms that explain obvious peripheral reasons for increased sensitivity) [31]. However, intra- and inter-subject response variability makes it difficult to derive clinically meaningful conclusions from individual QST measurements [30]. To improve upon this, researchers have developed a battery of individual QST measurements, but the series of tests is time consuming, requires specialized equipment, and needs specific expertise to interpret the results [30-33]. These features inhibit the implementation of individual, modality-specific QST in typical clinical practice settings, which prompted us to investigate other testing options.

Suprathreshold tests and multi-modal tests, such as the thermal and ischemic tolerance tests, are thought to be robust at detecting alterations in central sensitization. The problem is that the suprathreshold tests reported in the literature are time consuming and by nature produce a strong algesic response [2,3,11] – unfavorable characteristics for use in a clinical practice setting. For these reasons, we explored the stimulus properties produced by an electric toothbrush.

To our knowledge, no previous studies have investigated a multi-modal vibrotactile stimulus such as the one tested in our study in people with TMD pain. One study assessed the use of an electric toothbrush as a stimulus in people with burning mouth syndrome [34]. A statistically significant difference in pain intensity was found between pain and non-pain controls immediately following stimulation, and at 1 and 2 minutes after stimulation. This was similar to the observed response to heat and cold pain stimuli, which were also assessed; however, the magnitude of the group differences was largest for the vibrotactile stimuli compared to heat and cold pain at the 1 and 2 minute time points [34]. This finding is in line with the assumption that multi-modal stimuli produce a more robust response. This study did not report sensitivity, specificity, or test-retest data.

Several studies have investigated the individual components likely present within the multi-modal vibrotactile stimulus used in our study. It has been shown that the vibration detection threshold is impaired in TMD pain patients, such that larger amplitudes are needed to elicit a response [4]. Temporal summation of heat pain [3] and mild noxious mechanical stimuli [4] results in increased report of pain compared to pain-free controls. These reports suggest that TMD pain causes an overall increased sensitivity to varying types of superficial stimuli applied to the overlying facial skin [5]. It also demonstrates a lack of knowledge regarding the ability of various QST to discriminate TMD pain subjects from pain-free controls, since results from individual QST modalities are known to vary significantly [35].

At present, the most robust estimates of the sensitivity and specificity for diagnosing TMD pain have been derived within the RDC/TMD validation study, which has only been published in abstract form [36].

For the diagnosis of myofascial pain, the use of experienced gold-standard examiners produced a sensitivity ranging from 0.78 to 0.84 and a specificity ranging from 0.97 to 0.99 [36]. In the same study, blinded test examiners following the RDC/TMD protocol yielded a sensitivity ranging from 0.66 to 0.81 and a specificity of 0.90 to 0.92. This is similar to our values of 0.57 for sensitivity and 0.92 for specificity, which were derived from 5 minutes by applying a standard QST stimulus. It is desirable for a screening test to have a high specificity, thus protecting against false positives, even if this is at the expense of sensitivity [23]. Given that this test was applied in a population without long-term chronic pain or wide-spread pain, the application of a multi-modal stimulus using an electric tooth brush faired well against the gold-standard diagnostic test.

### Limitations of Findings

We investigated subjects with TMD pain because it is relatively common [37], diagnostically well defined [24,36,38], and known to be associated with increased central sensitization [2,3,5,6]. Since TMD pain is trigeminally-mediated, like other chronic orofacial pains, these patients often experience greater difficulty with routine oral care such as dental cleanings, root canal procedures, and tooth extractions [39,40]. Additionally, like all people with chronic pain conditions, patients with TMD pain are known to have more co-morbid psychological disorders [41] than pain-free persons, and those with elevated somatization tend to over-report physical symptoms [42]. At present it is not clear whether the changes observed with QST are the result of mechanisms associated with the chronic pain itself or co-morbid psychological disorders, as altered QST has been shown in depressed people without pain [43].

## Conclusion

In conclusion, this study showed that the screening technique under investigation – measurement of the pain response at over 60 seconds after application of vibrotactile stimulation with an electric toothbrush – has good specificity, modest sensitivity, and excellent test-retest reliability. The stimulus was accepted and tolerated by all subjects and has properties that are conducive for its use as a screening test in both clinical and population research. Further research is needed to better delineate the characteristics, limitations, and discriminative properties of this vibrotactile stimulus, as well as its potential clinical uses in prognostication and evaluation of treatment outcomes.

## Competing interests

The authors declare that they have no competing interests.

## Authors' contributions

DRN, AH, JOL designed the study protocol. AH performed the QST on all subjects. MTJ analyzed the data, with input from DRN. DRN and MTJ wrote the majority of the manuscript, with input from JOL, AH and ELS. Responses to referee's feedback and revisions to the manuscript were written by DRN, with advice and input in formulating the responses from ELS and MTJ. All authors read and approved the manuscript.

## Pre-publication history

The pre-publication history for this paper can be accessed here:


